# Unseen and untreated: implementing sarcopenic obesity care in Nordic general practice

**DOI:** 10.3389/fragi.2026.1815247

**Published:** 2026-05-22

**Authors:** Fereshteh Baygi, Peter Haastrup, Sussi Friis Buhl, Jesper Bo Nielsen, Charlotte Suetta, Michael Skovdal Rathleff, Jens Søndergaard

**Affiliations:** 1 Research Unit of General Practice, Department of Public Health, University of Southern Denmark, Odense, Denmark; 2 Department of Geriatric and Palliative Medicine, Copenhagen University Hospital Bispebjerg and Frederiksberg and Department of Clinical Medicine, Faculty of Health, University of Copenhagen, Copenhagen, Denmark; 3 Department of Health Science and Technology, Aalborg University, and Center for General Practice at Aalborg University, Aalborg, Denmark

**Keywords:** aging, diagnosis, Nordic countries, older people, sarcopenic obesity, treatment

## Abstract

**Background:**

Sarcopenic obesity (SO) is defined as the coexistence of low muscle mass and excess adiposity. Despite growing international attention, SO remains under-researched and under-recognized in Nordic countries, particularly in primary care, where body mass index (BMI)-driven approaches dominate and muscle health is rarely assessed.

**Objectives:**

The objective of this study is to discuss opportunities for integrating SO screening and treatment into everyday clinical care in primary care settings.

**Key insights:**

Limited prevalence data and inconsistent diagnostic criteria make SO difficult to detect in Nordic primary care settings. The increasing use of pharmacological weight-loss therapies further highlights the need for age- and muscle-informed approaches. Most studies are cross-sectional and conducted in hospitals/community samples, limiting insights into disease trajectories and intervention effects and widening the gap between research and clinical practice.

**Clinical relevance:**

Although muscle loss and obesity jointly worsen health outcomes, current obesity strategies rarely assess muscular health, creating a critical blind spot in clinical practice for older adults. Moreover, few clinical guidelines offer age-specific recommendations, limiting recognition and management of SO and contributing to suboptimal care for a growing older population.

**Conclusion and call for action:**

Nordic primary care represents a key setting for integrating SO case finding into existing preventive frameworks. We propose that primary care adopts pragmatic diagnostic pathways for SO based on a simple case-finding approach combining basic functional tests, nutritional risk assessment, and anthropometric measures alongside BMI, without additional resources. This should be supported by clinician education and cross-sector collaboration to ensure that both adiposity and muscle decline are addressed. Ultimately, bridging the gap between research and practice remains crucial to improving outcomes for older adults with SO.

## Introduction

Sarcopenic obesity (SO) is a clinical condition characterized by the coexistence of excess adiposity and sarcopenia, defined by low muscle mass and strength (Chen et al., 2025). Together, these two conditions form a syndrome that represents a serious yet often under-recognized threat to healthy aging and an increasingly invisible clinical and societal challenge in older adults. Sarcopenia and obesity can lead to severe functional impairments, often more than either condition alone (Prado et al., 2024). SO is associated with serious consequences, including physical disabilities, metabolic diseases, and even mortality (Benz et al., 2024; Silay and Selvi Oztorun, 2025). These findings are concerning because sarcopenia frequently remains undiagnosed; many older adults who appear well nourished may still have low muscle strength and mass, leaving them particularly vulnerable when excess adiposity is also present (Silay and Selvi Oztorun, 2025). Despite its consequences, it appears that SO is not routinely assessed in clinical settings (Prado et al., 2024). This lack of awareness leads to missed opportunities for appropriate management, which is essential for improving long-term outcomes, reducing hospitalization and long-term care needs, supporting healthy aging, and helping older adults remain independent. Importantly, SO is a potentially reversible condition, and current clinical management primarily focuses on resistance or multimodal exercise training combined with optimized energy and protein intake to reduce fat mass while preserving and improving muscle mass and strength (Habboub et al., 2025).

Despite increasing global attention to SO and its health implications (Yuan et al., 2022), SO remains insufficiently studied among older adults in the Nordic countries, particularly in Denmark. Our recent scoping review identified major gaps in the regional research, with most studies being small, cross-sectional, and applying inconsistent diagnostic criteria, leaving prevalence estimates weak (Baygi et al., 2024). Existing Nordic data suggest that SO exists in the older population: in Sweden, it was observed in 4% of women and 11% of men aged 75 years and older (von Berens et al., 2020), while a Finish study reported 5.8%–12.6% of adults aged 70 years and older at risk for developing SO, depending on obesity criteria (Sääksjärvi et al., 2023). In contrast, Denmark lacks published prevalence studies, highlighting a national evidence gap that contributes to SO remaining a blind spot in primary care, where body mass index (BMI)-driven approaches overshadow the inclusion of muscle health and functional capacity.

We consider the Nordic region a critical case as populations have longer life expectancies than those in the remaining parts of the world (Baygi et al., 2024), meaning that a larger proportion will reach ages at which sarcopenia and SO typically occur, thereby increasing the population-level burden. Therefore, the objective of this study is to argue for systematic integration of SO screening and management in routine clinical care for older adults.

Nordic countries share comparable welfare and healthcare structures, including publicly funded health systems (Stockmarr et al., 2016; Collaborators, 2019), and policy commitment to equitable, socially sustainable aging (Cuadrado et al., 2022). Therefore, conceptual and organizational lessons from one setting are often transferable within the region. To make the guidance actionable, we use Denmark as an exemplar based on our direct experience with the Danish healthcare system, to show how these strategies may be embedded in everyday clinical workflows, while acknowledging that region-wide application may require local or national adaptation.

## Current landscape

The current clinical management of SO in Danish primary care highlights a substantial gap in routine practice. Muscle health receives limited attention in everyday clinical work, and SO is therefore rarely identified, partly due to the lack of recognition of sarcopenia. This is driven by the continued reliance on BMI-based assessments, the absence of standardized diagnostic criteria in clinical workflow, and the lack of implementation of the existing ICD-10 code for sarcopenia in Denmark. As a result, the prevalence of SO remains uncertain; affected patients are not identified, and systemic documentation in primary care is hindered. These diagnostic challenges, combined with limited clinical awareness and the lack of clear diagnostic pathways, further reduce the likelihood that SO is recognized as a relevant condition during primary care consultations. This diagnostic gap is further amplified by limited emphasis on muscular health in preclinical medical education at universities, postgraduate general practitioner (GP) training, and preventive care, which traditionally prioritize cardiovascular disease, cancer, and other organ-specific conditions. These limitations may adversely affect even the highly structured Nordic healthcare system in which each citizen is registered—free of charge and based on geographic accessibility—with a designated GP (Haastrup et al., 2025) who is responsible for disease detection, risk factor assessment, long-term disease management, and follow-up.

Beyond diagnostic limitations, important gaps also exist in nutritional and therapeutic management. Current obesity treatment practices are primarily based on BMI, and they neither consider age nor assess muscle mass and muscle function before or during treatment. Such weight-centered approaches may fail to identify the co-existence of malnutrition and obesity in older adults. This dual burden often arises from diets that are nutrient-poor, particularly lacking sufficient proteins to meet physiological requirements (Barazzoni and Gortan Cappellari, 2020). Inadequate protein intake accelerates muscle loss and impairs physical function (Geirsdóttir and Pajari, 2023), and available data indicate that among Danish community-dwelling older adults above 80 years, most fail to meet the recommended protein intake of 1.2 g/kg/day outlined in Nordic nutrition guidelines (Buhl et al., 2022; Nordic Council of Ministers, 2023).

Physical inactivity further amplifies the risk because sedentary behavior is a well-established predictor of SO, morbidity, and mortality (Salinas-Rodríguez et al., 2025). Although local initiatives exist to promote physical activity, logistical and motivational barriers often limit participation among older adults (Larsen and Ryom, 2022). Moreover, older people frequently experience episodes of illness that require prolonged bed rest whether at home or during hospitalization, leading to accelerated loss of body weight and muscle mass, thereby increasing the risk of SO and its progression (Di Girolamo et al., 2021).

When lifestyle-based interventions are insufficiently addressed in routine practice, pharmacological treatment may introduce new challenges for older adults. The increasing use of incretin hormones including GLP1 and GIP hormones for weight reduction is a global trend (Kunutsor and Seidu, 2026), and this trend is also evident in Denmark (Mailhac et al., 2024). However, incretin hormone usage among older adults remains poorly characterized in the available data.

In Denmark, eligibility for pharmacological obesity treatment is primarily defined by BMI ≥30 or BMI ≥27 with at least one obesity-related comorbidity such as hypertension or hypercholesterolemia (Dansk, 2023). Although these criteria provide a standard framework for treatment initiation, they do not account for age-related considerations or incorporate assessments of muscle mass and function into clinical decision-making. This is particularly relevant given that intentional weight loss in older adults may accelerate muscle loss and contribute to functional decline (Reid and Bhasin, 2025). Despite these concerns, treatment is rarely combined with targeted nutritional and exercise interventions. This raises concerns about an emerging subgroup of patients with a post-GLP-1 SO, warranting the need for age- and muscle-informed approaches for obesity management (Prokopidis et al., 2025).

Finally, system-level barriers also play a significant role. Existing guidelines provide limited operational direction on how muscle health should be assessed or documented, and structured continuing education on SO for healthcare professionals remains scarce. In addition, the lack of standardized documentation and infrequent use of diagnostic coding systems restrict opportunities for surveillance, research, and quality improvement.

## Challenges and opportunities

### Diagnostic challenges

The optimal operationalization of diagnostic criteria for SO remains unresolved (Kemmler et al., 2017; Wei et al., 2023). Although some consequences of SO may be indirectly captured using commonly used geriatric assessment tools, such as the Clinical Frailty Scale (Rockwood et al., 2005), which is also used nationally in Denmark for frailty screening (Lietzen, et al., 2026), no validated diagnostic tools currently exist in primary care (Murphy et al., 2020), and interventions remain scarce and reactive, typically initiated after complications arise.

Moreover, current definitions often rely on body composition assessment using techniques such as DXA, CT, or bioelectrical impedance analysis (BIA) (Donini et al., 2022). These methods, however, are rarely available in primary care, creating a major barrier to routine identification and contributing to frequent underdiagnosis (Mondal and Bhattacharya, 2023). Consequently, older adults with SO are often misclassified as having obesity alone, leading to treatment decisions based solely on elevated BMI. This misclassification has important clinical implications. For example, GLP-1 receptor agonists may be initiated for weight management; however, in individuals with SO, rapid weight loss can accelerate muscle depletion, intensify functional decline, and cause or aggravate adverse outcomes (Donini et al., 2022; Prokopidis et al., 2025). These implications underscore the need for improved diagnostic precision in identifying SO in clinical practice.

### Preventive and management strategies

Limited awareness and understanding of SO among many healthcare professionals remain a major challenge (Mondal and Bhattacharya, 2023). Effective strategies for prevention, identification, and management in primary care and community settings are not yet fully established (Goisser et al., 2015). This lack of clarity is concerning given the well-documented consequences of muscle strength loss and excess adiposity (Ruiz et al., 2008; Baygi et al., 2024; Benz et al., 2024; Prado et al., 2024; Silay and Selvi Oztorun, 2025). Importantly, all older adults are susceptible to weight gain and progressive losses in muscle mass, strength, and physical function (Ruiz et al., 2008; Borba and Costa, 2025), often driven by inadequate or unbalanced nutrition and physical inactivity (Cederholm et al., 2017; Volkert et al., 2019; Donini et al., 2022).

Against this backdrop, general preventive efforts emphasize maintaining muscle health and supporting weight stability. National and international guidelines consistently recommend optimal physical activity, structured exercise, and adequate dietary intake (Deutz et al., 2014, Volkert et al., 2019; Bull et al., 2020; Ahrensberg and Petersen, 2023; Blomhoff et al., 2023). These recommendations offer a clear opportunity to mitigate SO by leveraging existing evidence on lifestyle behaviors. However, translating guidelines into practice remains challenging. Effective implementation requires sufficient allocation of healthcare professionals’ time and resources, as well as tailored and consistent communication to and between healthcare professionals to ensure that the relevance of these recommendations is well understood and widely shared (Thoonsen et al., 2024). In addition, supportive environments and care settings are needed to enable older adults to remain physically active, including access to physical activity through services and programs tailored to the needs, preferences, and goals of all individuals with SO (World Health Organization, 2023).

### System-level opportunities

Denmark’s well-developed primary care system offers considerable opportunities to strengthen prevention and early identification of SO. Key strengths include skilled healthcare professionals—GPs, practice staff, and municipal multidisciplinary teams—supported by a long-standing tradition of nationally regulated preventive care and early risk detection (Sundhedsstyrelsen, 2020). Existing frameworks offer clear entry points for integration. However, we believe that effective collaboration between sectors remains challenging, limiting the potential impact of existing frameworks and weakening coordinated prevention and care pathways.

## A new perspective

Current obesity strategies, dominated by body weight and BMI, obscure early muscle loss and functional decline in older adults, delaying recognition of SO and consequently increasing the risk of inappropriate treatment.

We propose a shift from BMI-centered obesity management toward a pragmatic muscle-informed approach to SO in primary care. Rather than positioning SO as a condition dependent on specialist diagnostics, we argue that it should be understood as a clinically relevant syndrome aligned with the core responsibilities of primary care. SO reflects the convergence of excess adiposity, declining physical function, and nutritional vulnerability—domains already familiar to GPs. The central challenge is therefore practical: embedding these dimensions into routine, feasible assessment processes.

Accordingly, we move beyond exclusive reliance on BMI and propose multidimensional case-finding that integrates simple measures of muscle function, nutritional risk, and physical performance, alongside traditional indicators of adiposity. Although diagnostic consensus remains limited, we reframe this uncertainty as a rationale for early identification of individuals at risk, rather than a justification for inaction. Emphasizing functional consequences and clinical risk profiles enables timely intervention without dependence on strict diagnostic thresholds.

This perspective also addresses unintended consequences of contemporary obesity management in older adults, particularly the risk of accelerated muscle loss associated with pharmacological weight-loss therapies when muscle health is not assessed. A muscle-informed approach supports safer, more individualized treatment by aligning weight reduction with nutritional optimization and exercise-based interventions.

Finally, the proposed perspective is explicitly implementation-oriented. Existing Nordic primary care structures, routine health checks, and collaboration with municipal preventive services provide concrete entry points for integrating functional and nutritional assessment into everyday workflows.

## Implications for practice, research, and policy

### Practice

Routine health checks in Danish general practice often include BMI, blood pressure, HbA1c, and individualized risk assessments. Expanding these consultations to incorporate indicators of nutritional status and functional decline (e.g., basic physical function tests) could facilitate earlier recognition of individuals at risk of SO and support timely identification and preventive management (Kristensen et al., 2022). Similarly, Danish municipalities are mandated to provide preventive services for older adults, including structured home visits (Eldrelov, 2024), which offer a unique opportunity for screening and supplying information about optimal physical activity and nutrition. Leveraging these established systems, combined with practical screening tools and clear clinical pathways, presents a significant opportunity to improve early detection and targeted management of SO. Although GPs increasingly implement ultrasound into routine clinical care (Andersen et al., 2022), BIA remains a faster and more feasible option in primary care. Ultrasound-based muscle assessments such as sarcopenia echography (SARCHO) have shown good validity for diagnosing sarcopenia (Brockhattingen et al., 2025) and may, in the future, also prove useful for assessing SO. Combined ultrasound-derived measure with simple anthropometric indicators of adiposity could represent a promising complementary approach, particularly where access to DXA is limited. However, because the validity of ultrasound for diagnosing SO has not yet been established and higher adiposity can make ultrasound assessment technically challenging, it is currently best considered a potential future complementary rather than a primary diagnostic tool.

### Research

Evidence on the early detection and management of SO remains limited. However, evidence from related conditions, such as sarcopenia, reduced physical function, and frailty, shows that interventions involving progressive resistance training, alone or combined with high-protein diets, can effectively improve muscle strength, power, and mass (Cadore et al., 2014; Hvid et al., 2016; Liao et al., 2019; Huang et al., 2021). Importantly, these approaches have proven feasible in Danish primary care settings (Hvid et al., 2016; Olsen et al., 2022). Future research should focus on adapting these strategies for SO, evaluating their effectiveness, and exploring cost-effectiveness and implementation models. Obesity is associated with sociodemographic factors (Magnusson et al., 2014), but whether SO follows a similar social gradient in the Nordic countries remains, to the best of our knowledge, unknown. Evidence from other regions suggests this to be the case (Nishioka and Avan, 2025), warranting further research to guide prevention and management strategies, support knowledge dissemination, and inform subsequent implementation in other Nordic countries.

### Policy

Policy support is essential to translate emerging approaches to SO identification and management in routine practice. To achieve this, policy measures should facilitate cross-sectoral integration, strengthen preventive care, and enable effective data sharing between GPs and municipalities. In addition, the adoption of emerging methods for muscle health assessment (e.g., ultrasound and anthropometrics) (Maffini et al., 2024; Brockhattingen et al., 2025) requires strong policy support. Embedding practical screening tools into clinical guidelines alongside training programs for healthcare professionals will be key to ensuring timely detection and sustained implementation.

### Future directions

The existing multi-sectoral infrastructure in the Nordic countries, availability of multidisciplinary professionals, and evidence from other age-related conditions provide strategic advantages for improving SO management in primary care. First, routine health checks and municipal preventive initiatives provide opportunities to raise awareness about physical activity and nutrition and to enable early recognition of older adults at the risk of SO. Second, continued education and knowledge updates within current frameworks can strengthen healthcare professionals’ capacity to identify and manage SO. Third, municipal facilities and personnel are well positioned to deliver targeted intervention, such as integrated exercise and nutritional programs, which take into account the physical, psychological, and social circumstances of older adults, in line with current recommendations (Izquierdo, de Souto Barreto et al., 2025), offering considerable potential to address SO across wider aging populations.

Fourth, national clinical guidelines can support integrated prevention and management pathways. Realizing these opportunities will require coordinated cross-sector efforts, research to validate practical screening tools, and implementation strategies to embed SO care into routine practice.

## Conclusion

SO remains a growing yet underrecognized condition among older adults in Denmark. Ongoing diagnostic uncertainty and limited awareness hinder timely detection and effective management, while preventive initiatives largely remain oriented toward single-disease conditions, leaving SO insufficiently addressed. We propose that, despite these challenges, early identification of individuals at risk should not be postponed. Rather, SO should be conceptualized as a clinically relevant risk constellation, in which functional decline and nutritional vulnerability provide a practical basis for timely intervention. Importantly, this approach can be embedded into existing general practice consultations, including annual chronic care reviews and preventive health checks, without requiring major structural changes to current workflows.

Existing preventive frameworks and interventions offer clear opportunities for action. Realizing this potential will require the development and implementation of pragmatic diagnostic tools, routine screening, and targeted interventions. A shift toward cost-effective case-finding in high-risk populations, combined with integrated management linking GP-led detection with dietician-guided nutritional strategies and municipal awareness, is urgently needed to mitigate the adverse outcomes associated with SO and promote healthy aging.

## Data Availability

The original contributions presented in the study are included in the article/supplementary material, further inquiries can be directed to the corresponding author.
